# Diagnosis and surgical management of a type IVb dermoid sinus between the parietal and occipital bones in a young cat

**DOI:** 10.1177/20551169261478735

**Published:** 2026-08-05

**Authors:** Sophie E. Fish, Anna Adrian, Kaspar Matiasek, Lara Matiasek

**Affiliations:** 1 Small Animal Referral Centre, Frontier Veterinary Specialists, Munich, Germany; 2 Section of Clinical & Comparative Neuropathology, Centre for Clinical Veterinary Medicine, LMU Munich, Germany; 3 Small Animal Clinic, Centre for Clinical Veterinary Medicine, LMU Munich, Germany

**Keywords:** intracranial cyst, epidermal inclusion, brain surgery, CT, MRI, neuropathology

## Abstract

**Case summary:**

A dermoid sinus is a congenital, tubular skin defect caused by incomplete separation of the skin and nervous system during embryonic development. It is more common in dogs and usually located along the spine. Intranasal or superficial cranial forms have been occasionally reported. This case describes a dermoid sinus on the head of a cat with intracranial extension, illustrating diagnostic and surgical challenges. A 1.5-year-old, female British Shorthair cat was evaluated for a recurring, previously superficially resected, cutaneous mass on her head with an abnormal pattern of hair growth. MRI and CT revealed an extra-axial mass extending in a tubular fashion through a midline defect between the parietal and occipital bones, continuing caudally along a malformed tentorium. Mild focal cerebellar compression and suspected secondary meningitis, more evident on the right side, were also identified. Surgery was performed via a combined caudotentorial and limited right-sided rostrotentorial craniectomy. Careful dissection along the lesion margins and durectomy of focally attached thickened meninges allowed complete resection. No postoperative complications or further neurological deficits occurred. Histopathology confirmed a dermoid sinus. Follow-up after 28 months revealed no recurrence.

**Relevance and novel information:**

To the authors’ knowledge, this is the first reported case of a type IVb dermoid sinus in a cat to be successfully managed surgically. This case provides important clinical information regarding the diagnosis, imaging features and surgical management of complex intracranially extending dermoid sinuses in feline patients.

## Introduction

A dermoid sinus is a tubular skin defect resulting from incomplete separation of the skin and nervous system during embryonic development, representing a non-occurring dorsal disjunction phenomenon.^1^ It can arise anywhere along the neuroaxis. The depth of the tract varies and may be confined to the subcutaneous tissue or extend to the dura mater.^2–8^ It is most frequently seen as a heritable condition in Rhodesian Ridgebacks^9–11^ and has also been described in other dog breeds.^4,5,12–14^ In cats, dermoid sinuses are rare and have been reported primarily in the cervical spine.^15^ Previous case reports identified cranial dermoid sinuses mostly in the nasal region with intracranial extension in several dogs^3,6,9,16–18^ and a cat.^8^ One case report detailed a type IVb dermoid sinus that contacted the meninges and extended through a defect in the frontal bones.^17^ To date, no non-nasal dermoid sinus with intracranial extension has been reported in a cat.

This case report describes associated diagnostic and surgical challenges of a dermoid sinus with intracranial extension in a British Shorthair cat.

## Case description

A 1.5-year-old, female British Shorthair cat was presented for a recurring mass on the top of her head (Figure 1), which had been present since 12 weeks of age. It had been superficially resected twice, 16 and 4 weeks earlier. On both occasions, regrowth occurred shortly after. No diagnostic imaging had previously been performed.

**Figure 1.**
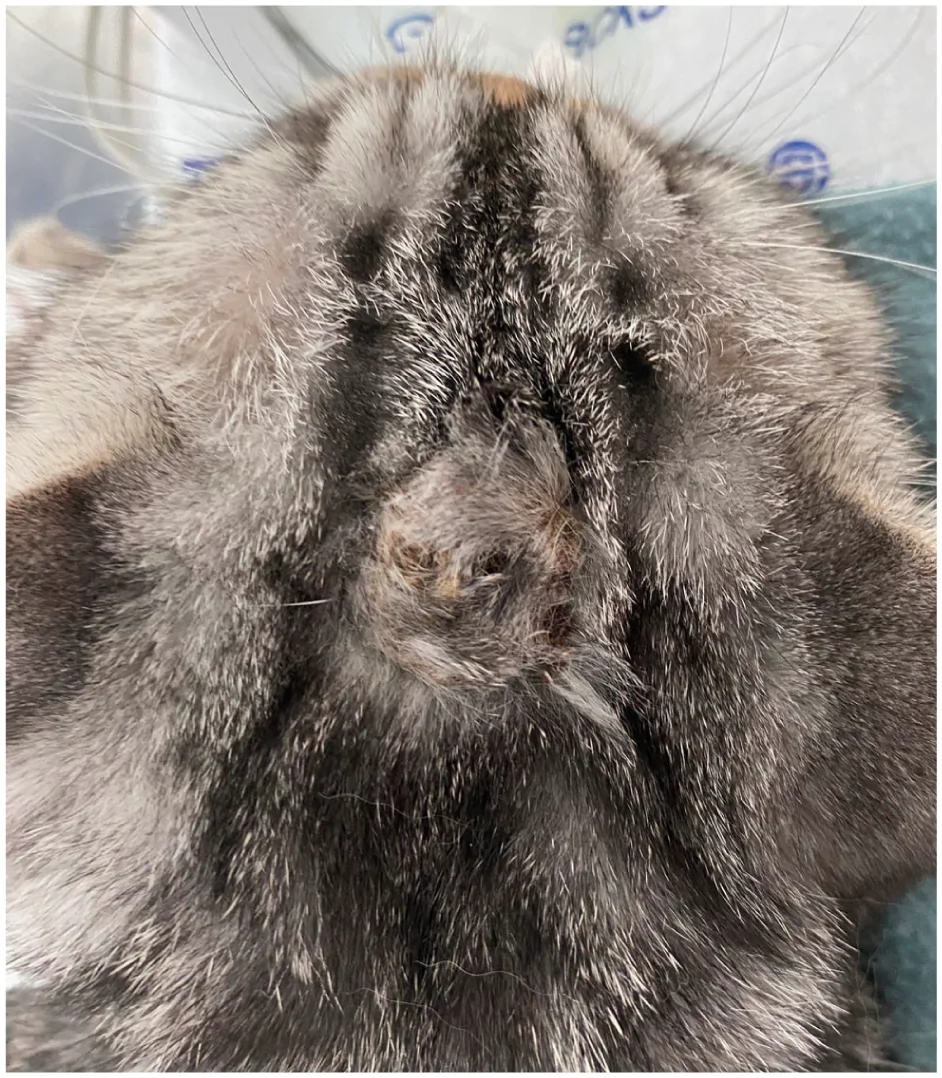
The patient’s head before shaving: a round, elevated, non-painful mass on the top of the head with an abnormal pattern of hair growth and small ulcerations leading to a crusty skin flap is evident.

Upon presentation, the owners reported pruritus at the lesion site. The mass was round, elevated and non-painful, with an abnormal pattern of hair growth and small ulcerations that had formed a crusted flap (Figure 1). Clinical examination was otherwise unremarkable. Neurological examination revealed mild upper motor neuron (UMN) proprioceptive deficits in the hindlimbs (left more than right) and the left forelimb.

MRI was performed using a 1.5 T scanner (Magnetom Altea; Siemens). Sequences included a sagittal T2-weighted sequence of the head and cranial cervical spine to C2, and transverse T2-weighted, fluid attenuation inversion recovery, and T1-weighted pre- and post-contrast images of the head. Within the subcutaneous tissue, overlying the caudal parietal to cranial occipital bone, a hyperintense structure with focal fluid accumulation, considered as a possible abscess, was identified (Figures 2 to 4). A thin band extended into the cranium, and the occipital bone appeared heterogeneous at this level. Both the bone and underlying meninges showed moderate contrast enhancement (Figures 3 and 4). The venous dorsal sagittal and transverse sinuses could be traced to the lesion; however, no further identification at the lesion site itself was possible. Focal compression of the cerebellum was noted rostrodorsally (Figure 4). The cerebellum was caudoventrally displaced towards the foramen magnum.

**Figure 2.**
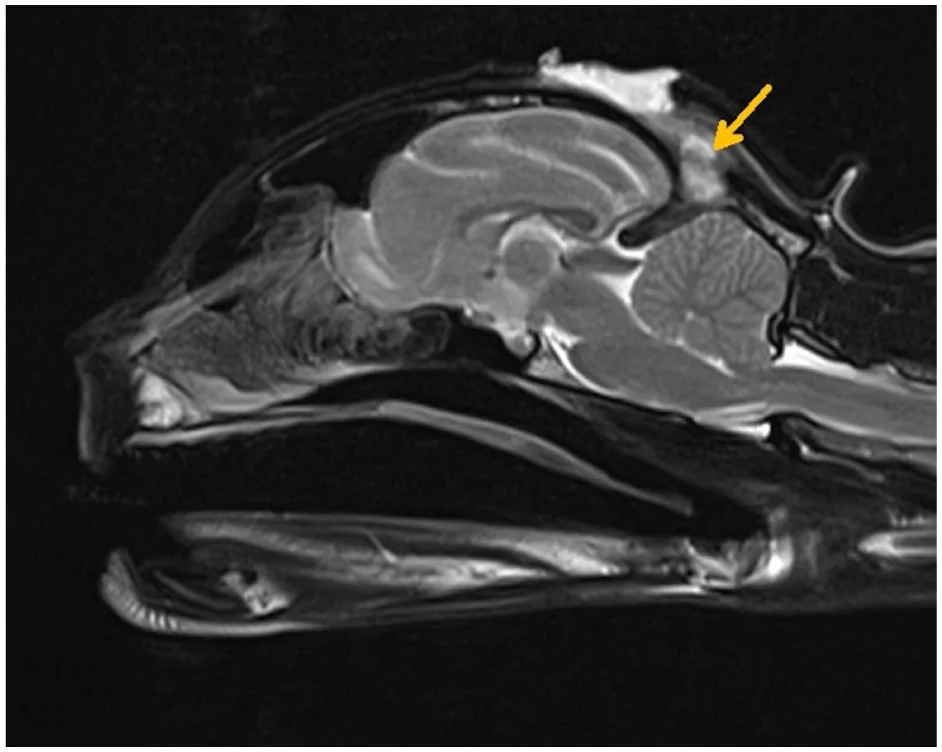
Sagittal T2-weighted MRI: extra-axial mass extending in a tubular fashion through a midline defect between the parietal and occipital bones, caudally along the malformed tentorium (arrow).

**Figure 3.**
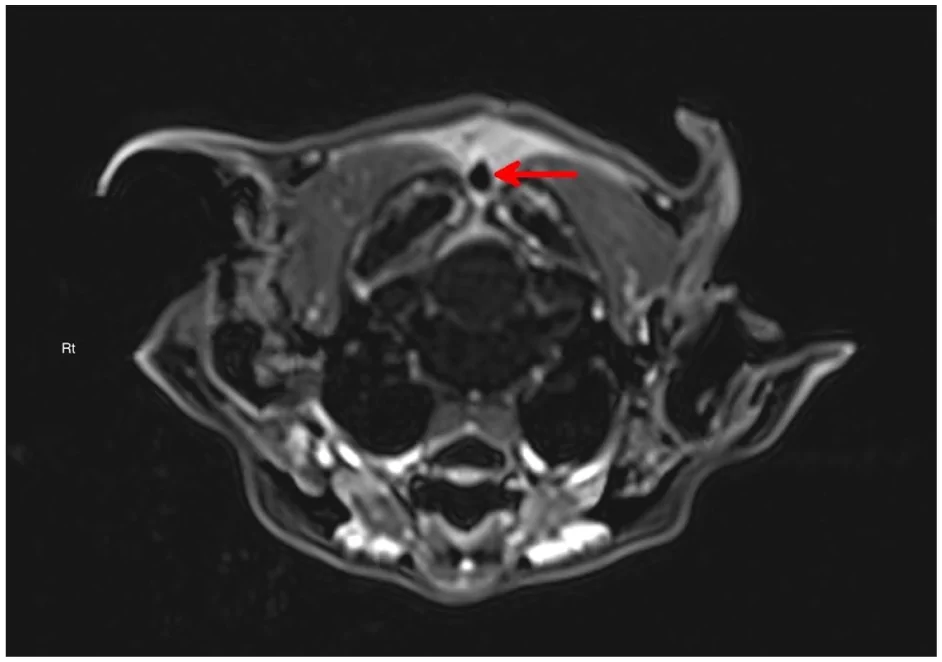
Transverse T1-weighted subtraction MRI at the level of rostral cerebellum: suspected abscess (arrow) with secondary meningitis.

**Figure 4.**
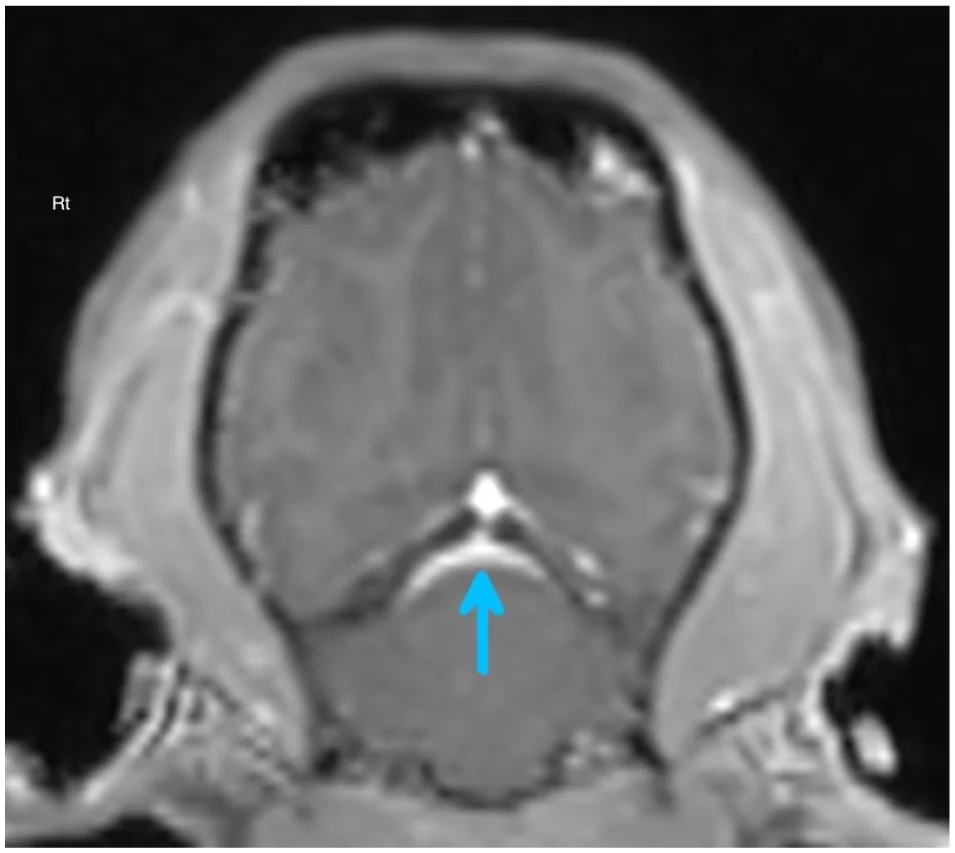
Dorsal T1-weighted post-contrast MRI: mild focal cerebellar compression and suspected secondary meningitis at the rostral border of the cerebellum (arrow), slightly more right-sided.

Clinical and MRI findings were most consistent with a dermoid sinus extending intracranially. There was suspicion of abscess formation and secondary meningitis.

To further evaluate the osseous structures for surgical planning, CT was performed using a 128-slice scanner (Somatom Definition Edge; Siemens). The tentorial bone was fused only at its rostral end, leaving a small midline gap (Figure 5). No bone was present on the caudal aspect of the tentorium where it transitions to the occiput, resulting in a midline defect slightly extending to the right (Figures 5 and 6). The suspected dermoid sinus was expanding through this region. The CT examination of the entire cervical spine was unremarkable.

**Figure 5.**
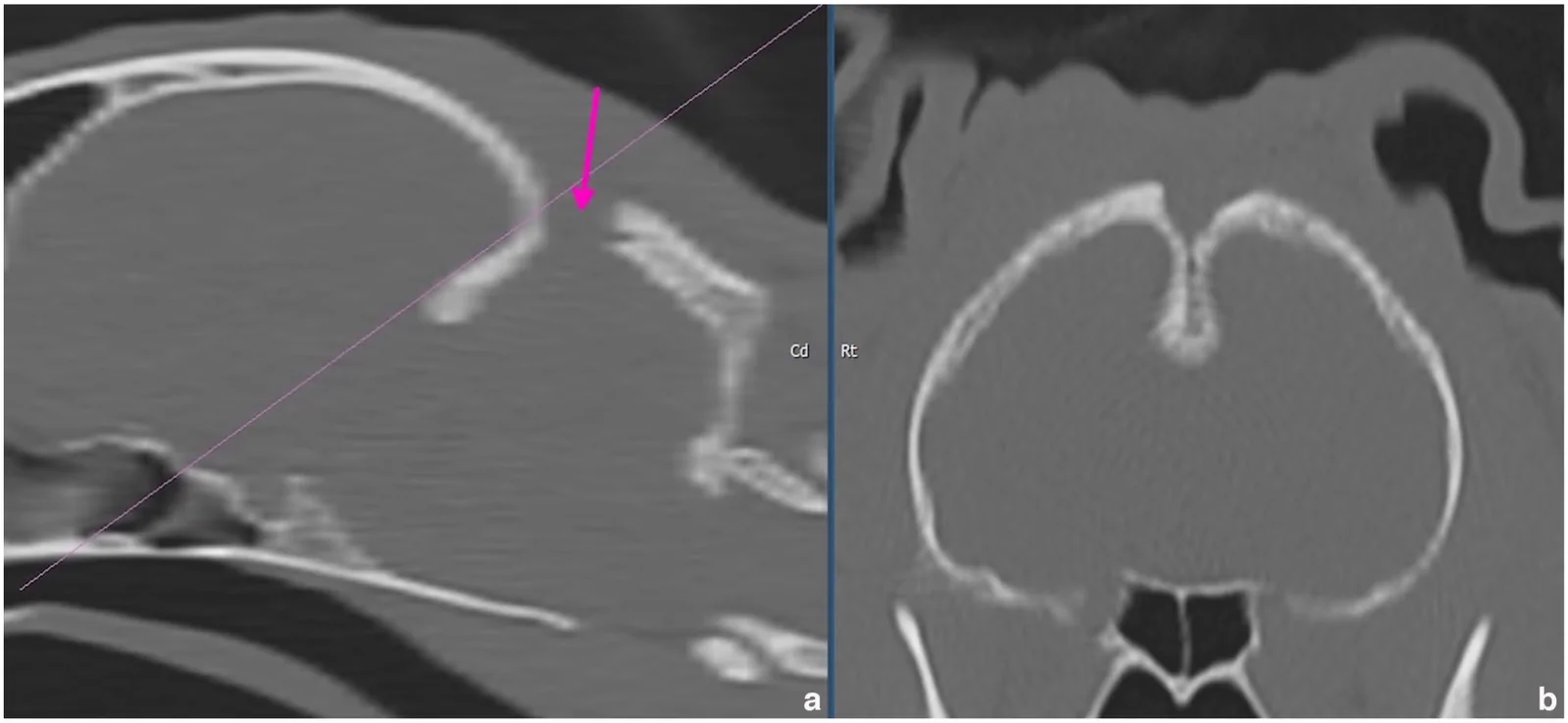
Sagittal multiplanar reconstruction of (a) the CT image showing a midline gap at the tentorial bone where it transitions to the occiput (arrow) and (b) transverse CT through the malformed osseus tentorium cerebelli.

**Figure 6.**
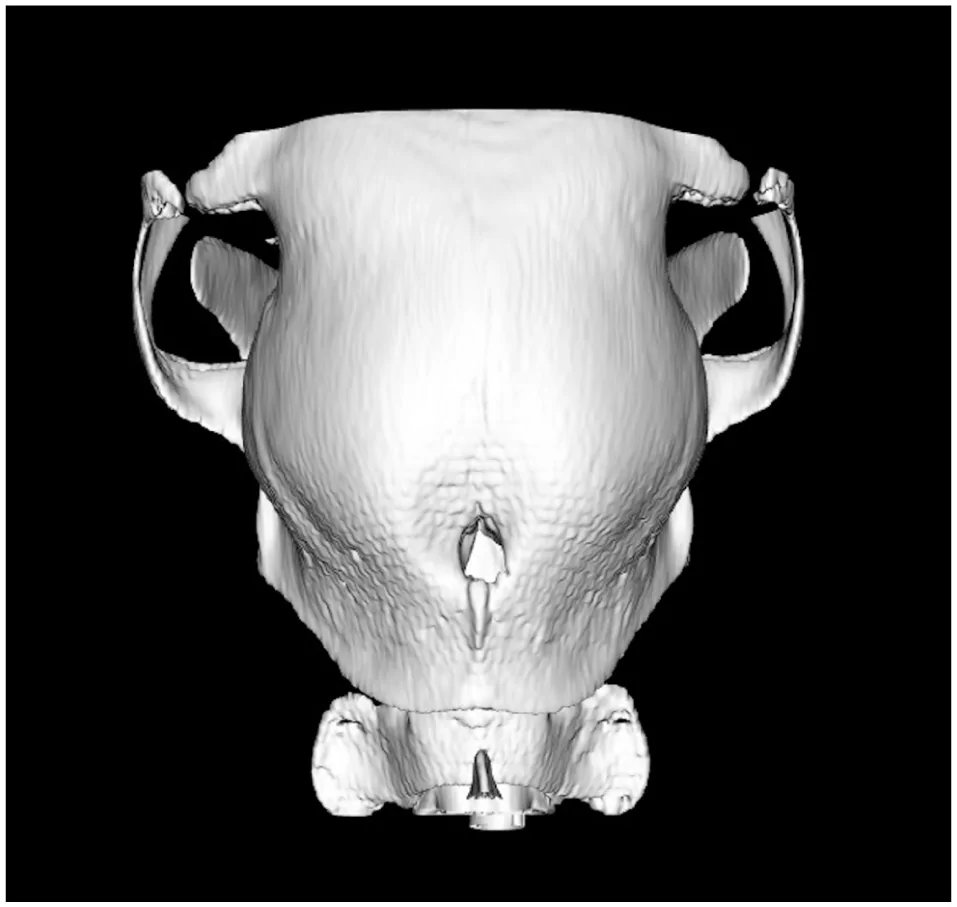
Three-dimensional reconstructed CT showing the bony midline defect between the parietal and occipital bones, slightly extending to the right.

The owners elected for surgery. During clipping, the crusted flap detached from the skin, exposing subcutaneous tissue at the centre of the mass (Figure 7). An ellipsoid incision was made around the lesion, extending caudally to the occipital protuberance. Dissection around the mass exposed its sinusoidal extension into the osseous defect. A bilateral mid-caudotentorial and limited right-sided rostrotentorial craniectomy was approached around the sinusoidal lesion (Figure 8). The defect was widened using a Kerrison rongeur before careful dissection rostrally, caudally, laterally and in depth between the cerebellum and the osseus tentorium cerebelli to expose its extension further (Figure 9). Neither the venous dorsal sagittal nor transverse sinuses were encountered. The sinusoidal lesion was removed, followed by a durectomy of highly thickened meninges above the cerebellum. The rostral aspect of the cerebellum showed a bluish-reddish discoloration; otherwise, the neuroparenchyma was unremarkable. No visible remnants of the suspected dermoid sinus remained (Figure 10). The surgical site was carefully lavaged, and the craniectomy defect was covered with Lyostypt (Braun), a compress from native collagen of bovine origin, for local haemostasis. The wound was closed in two layers by advancing the skin cranially using intradermal sutures to cover the defect (Figure 11).

**Figure 7.**
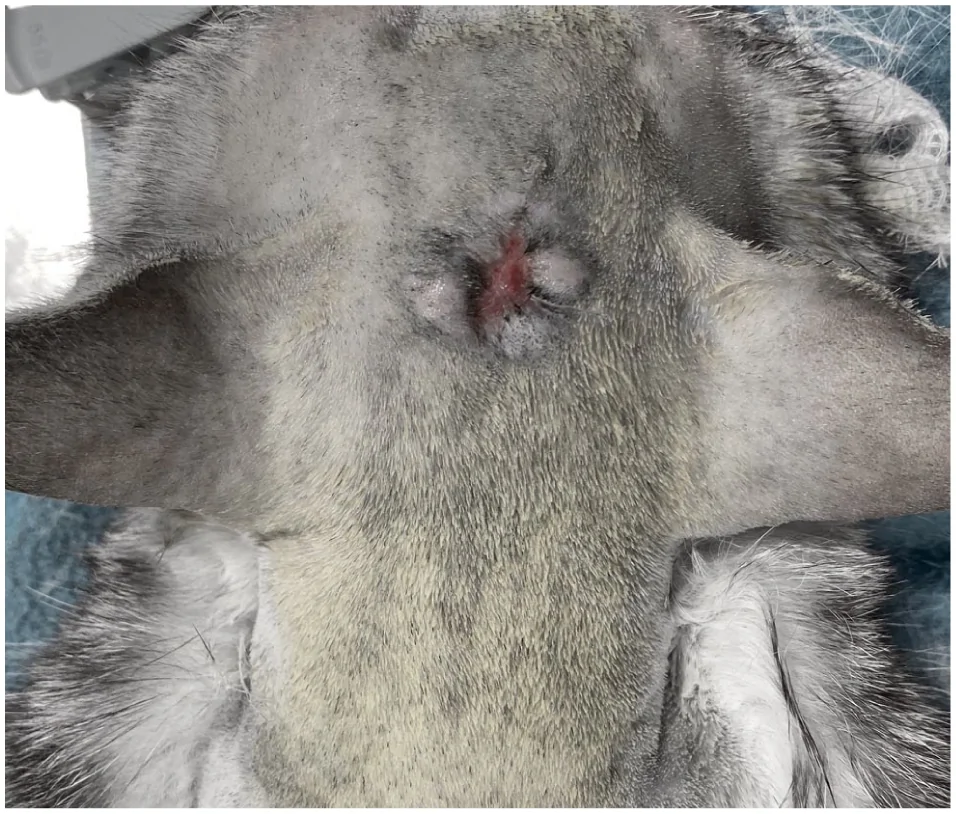
The patient’s head after shaving: the crusty flap had fallen off, showing subcutaneous tissue in the centre of the mass.

**Figure 8.**
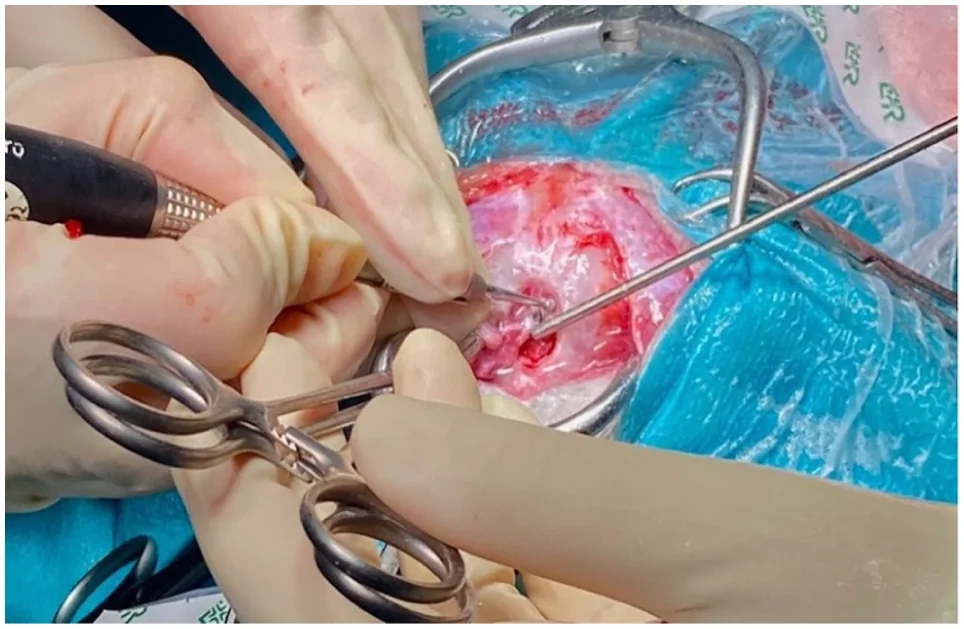
Bilateral mid-caudotentorial and limited right-sided rostrotentorial craniectomy revealing the sinusoidal extension.

**Figure 9.**
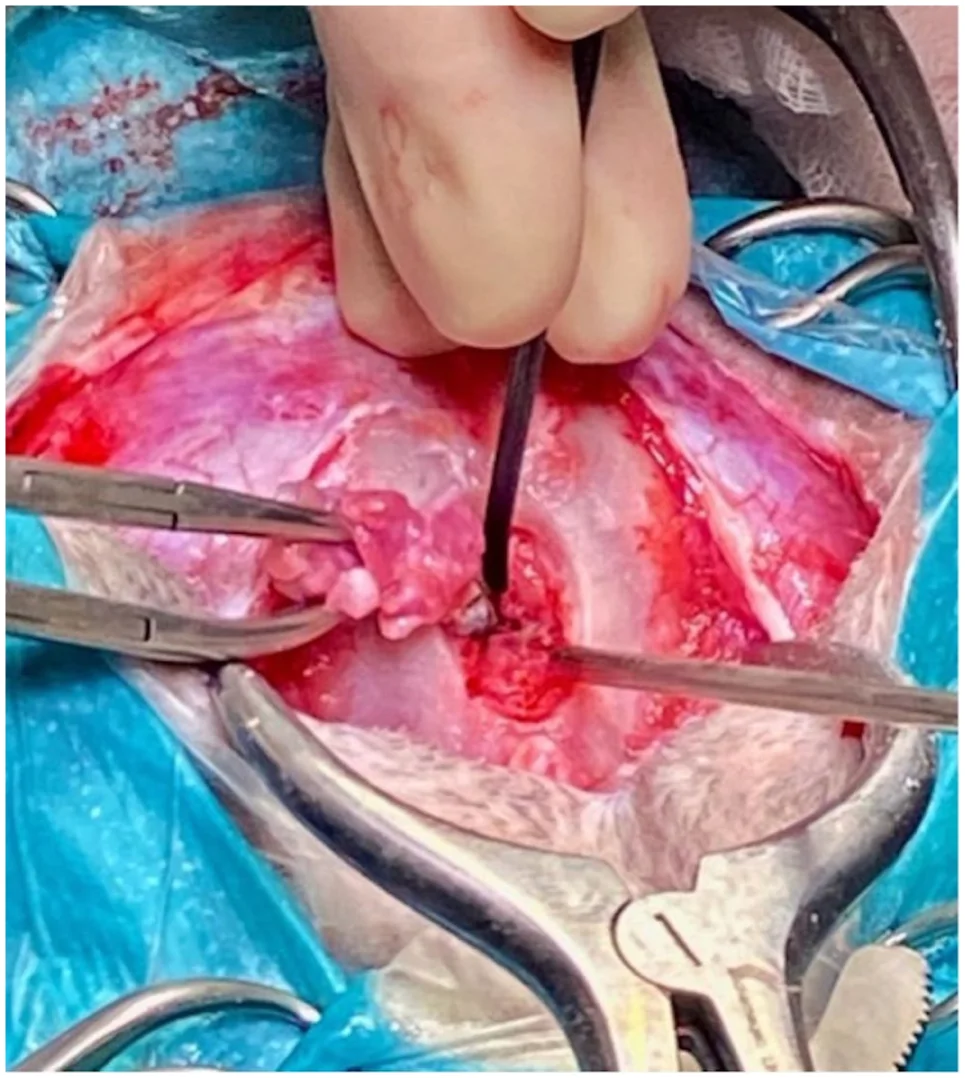
Widening of the defect around the sinus with a Kerrison rongeur before careful dissection around the sinus towards the brain.

**Figure 10.**
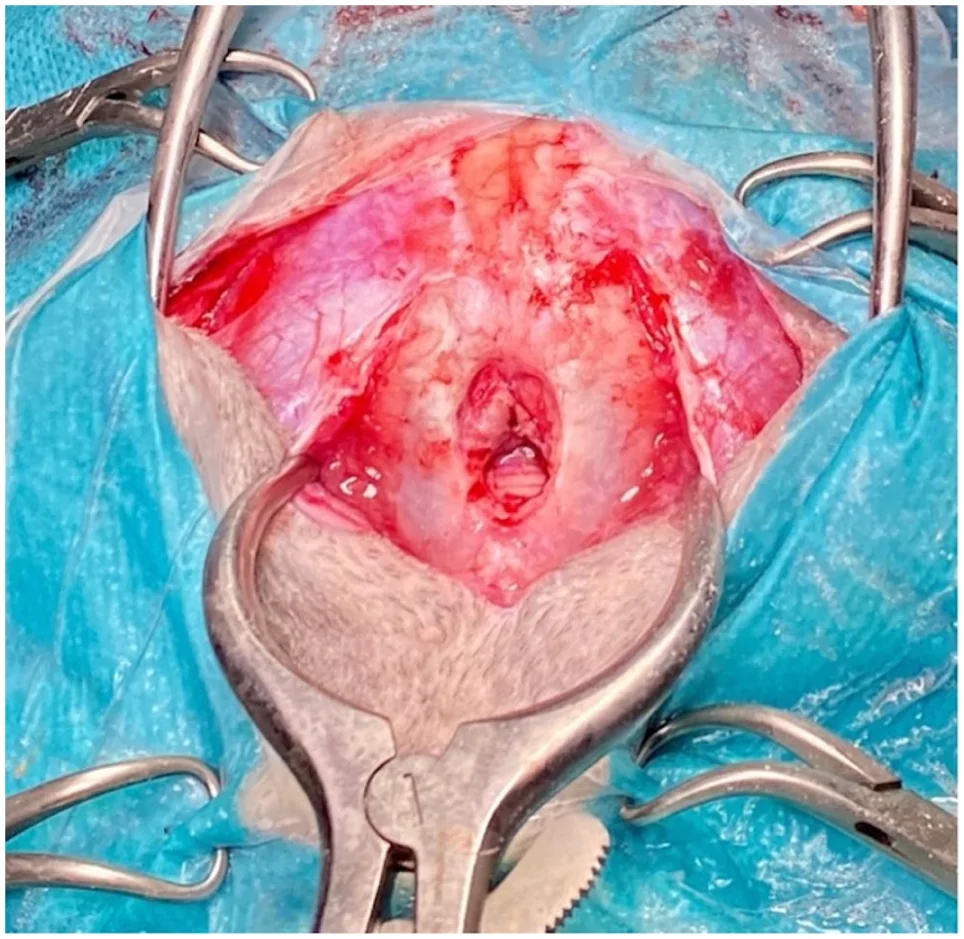
The rostral part of the cerebellum showed a bluish-reddish discoloration; otherwise, the neuroparenchyma was unremarkable. No visible remnant of the suspected dermoid sinus was left in situ.

**Figure 11.**
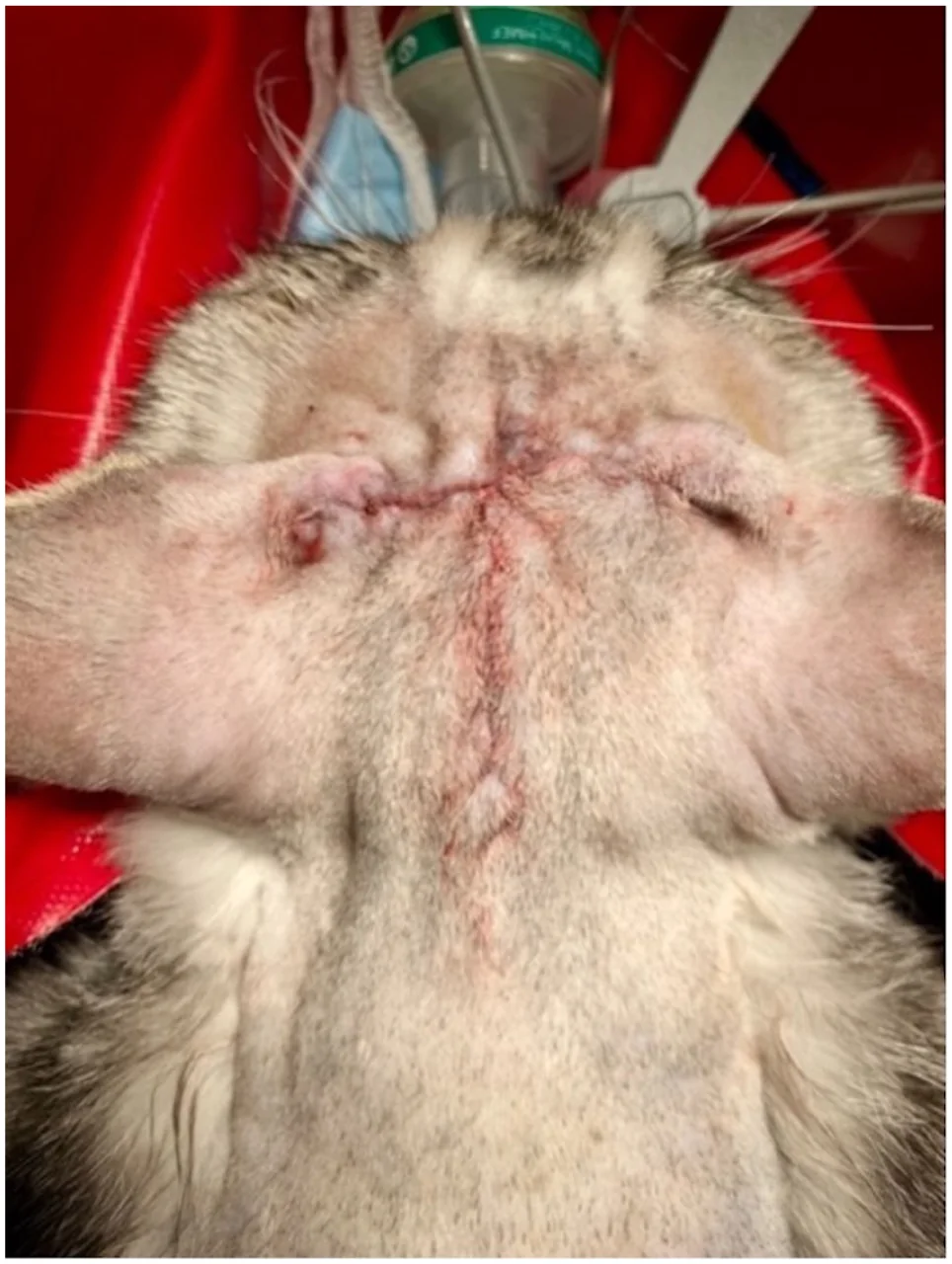
After completed wound closure.

The excised mass and crusted flap were submitted for histopathological examination. The flap consisted entirely of necrotic tissue. The skin surface over the mass was partially ulcerated and covered by serocellular crusts, with a central dome-shaped area of scar tissue. This tissue connected to a strand of fibrocollagenous tissue that extended deeply between muscle fascicles and was surrounded by fibrovascular granulation tissue containing numerous trapped, fragmented hairs and hair bulbs, along with other dysplastic adnexal structures. Within the centre of the granulation, an epithelial-lined duct filled with keratin lamellae arose from a highly pigmented and moderately hypergranulated keratinising squamous epithelium. The duct was enclosed by a thick fibrocollagenous capsule. At the periphery of the granulation tissue, mixed polymorphonuclear, histiocytic and plasmacytic infiltrates were present, tapering towards the biopsy margins. No intralesional microorganisms were seen. This mass resembled a dermal sinus embedded in a bulk of tissue affected by secondary inflammation and formation of scar tissue (Figure 12). Based on the histopathological margins, the mass was fully excised. There was no indication of deep bacterial infection.

**Figure 12.**
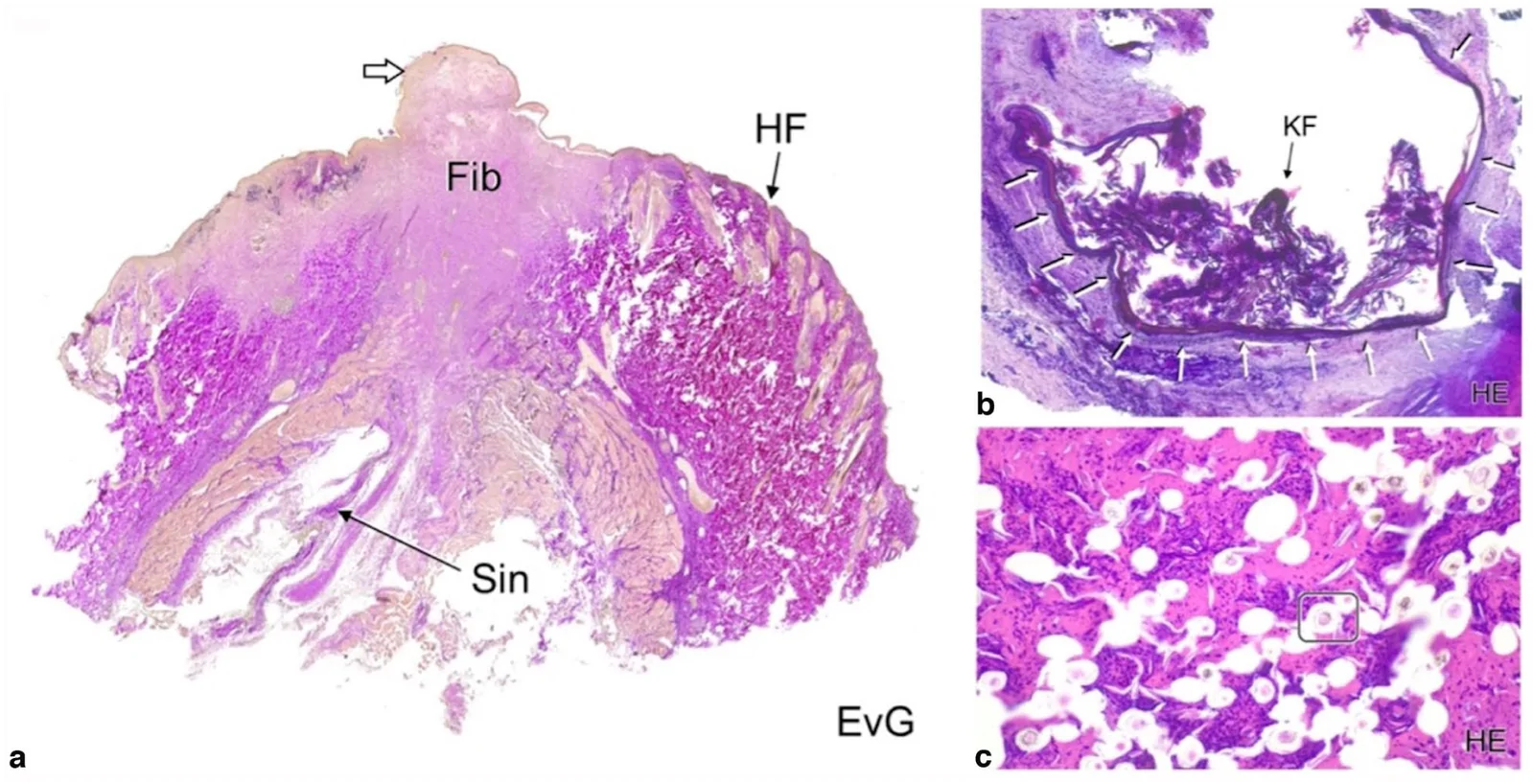
(a) Histological section along the course of the sinus. Note the thickened skin with fibrotic scar tissue in the centre and the sinus extending into the depth. (b) Closer view of the sinus wall and contents. The centre is filled with keratin flakes (KF) arising from a peripheral lining of stratified squamous epithelium (arrows). (c) Where hairs (frame) came into contact with the surrounding mesenchymal tissue, they incited an exsudative necrosuppurative inflammation. *Note.* EvG: Elastica van Gieson stain; Fib: dermal fibrosis, HE: haematoxylin and eosin stain; HF: hair follicle; Sin: sinus tract.

Clinical, imaging and histopathological findings were consistent with a type IVb dermoid sinus between the parietal and occipital bones.

Postoperative care included 4-day hospitalisation with antibiotics (cefazolin 22 mg/kg IV q8h for 3 days, then transitioned to oral) and dexamethasone (0.2 mg/kg IV q24h for 2 days, then changed to prednisolone on day 3: 1 mg/kg PO q24h, day 4: 0.7 mg/kg q24h). Neurological status was monitored via modified Glasgow Coma Scale.^19^ The postoperative course was uneventful, with no complications or new neurological deficits. The cat was discharged in good condition with prednisolone (0.7 mg/kg q24h, tapering after 2 weeks) and cefazolin (15 mg/kg q12h for 2 weeks).

At recheck examinations 1 and 2.5 weeks postoperatively, the cat was doing very well. At telephone follow-up after 11 (Figure 13), 20 and 28 months, the owner reported no regrowth or other clinical abnormalities.

**Figure 13.**
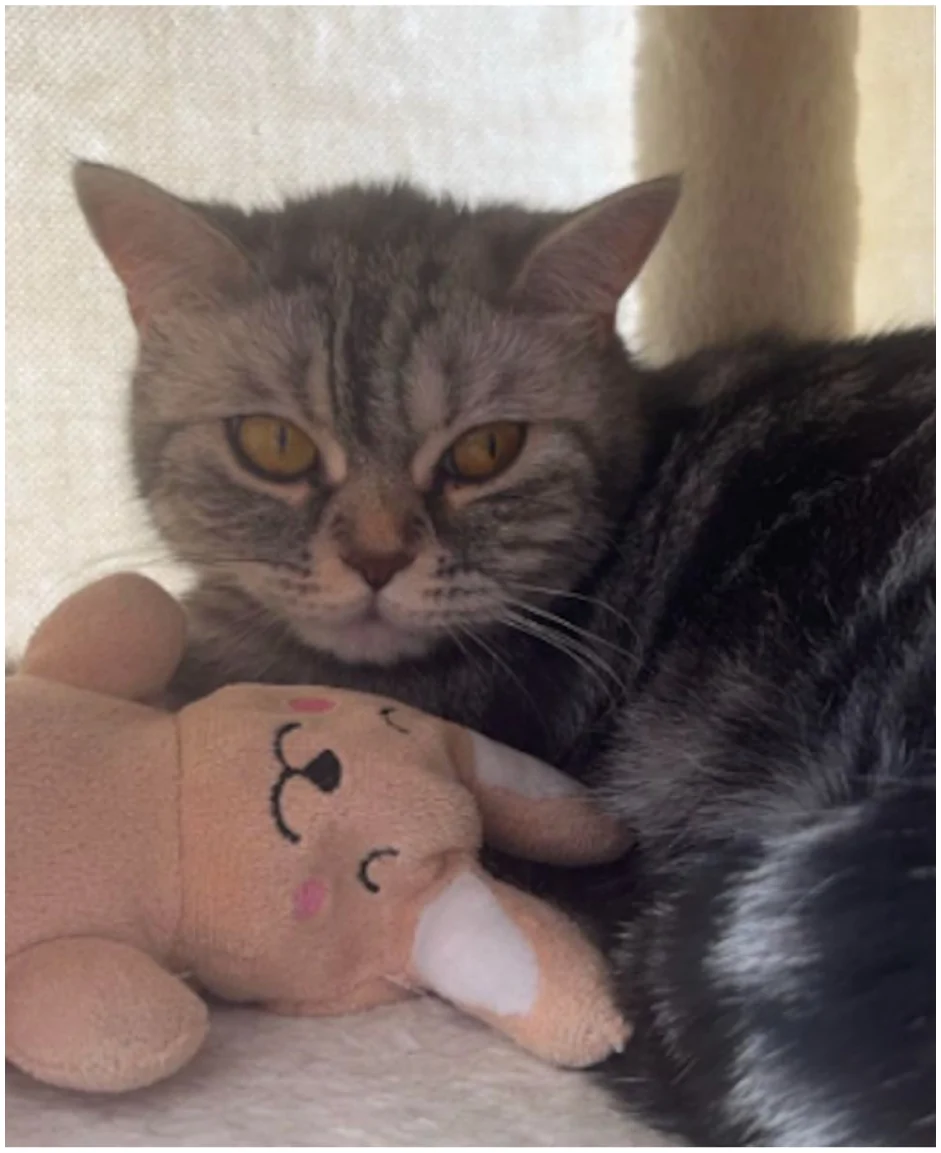
Eleven months postoperatively.

## Discussion

This case report describes a rare type IVb dermoid sinus with intracranial extension located between the parietal and occipital bones of a young British Shorthair cat. Although dermoid sinuses are mostly seen in the cervical and thoracic region of specific dog breeds (e.g. Rhodesian Ridgeback),^10,11^ they are exceptionally rare in cats, especially with intracranial extension.

Correct classification of a dermoid sinus informs the complexity of surgical management and is paramount to successful treatment, as the involvement of the dura or an intracranial extension may significantly complicate treatment. Dermoid sinuses have traditionally been classified according to their depth and relationship to the supraspinosus ligament.^10^ They were defined as tubular blind sacs located along the dorsal midline and extend from the skin ventrally into the underlying tissues.^10^ Types I–III vary in depth, but do not reach beyond the supraspinous ligament. Type IV sinuses extend deeper, into the dura mater. Additional types have since been proposed: type V is a closed epithelial cyst,^5^ whereas a type VI presents as an open sinus tract‭ extending to‬ the level of the supraspinous ligament with a distal connecting strand to the dura mater.^20‬‬‬‬‬‬‬‬^

The abovementioned classification relates to the vertebral column only. To address dermoid sinuses according to their anatomical locations, three subtypes were proposed: subtype a for dorsal midline sinuses along the spine; subtype b for those on the head (excluding the nose); and subtype c for nasal dermoid sinuses.^6^ A recent review exclusively describes dermoid cysts in cats, mostly focusing on the cervical area.^21^

In the present case, and in particular owing to the atypical location of the dermoid sinus, advanced imaging was prerequisite for diagnosis and surgical planning. MRI identified a hyperintense tubular structure with a focal fluid accumulation, possibly representing an abscess. However, histopathology revealed sterile inflammation without abscess formation or microorganisms. Therefore, antibiotic treatment was discontinued after the histopathology report revealed no bacterial involvement.

CT provided critical additional information regarding the osseous defect that permitted intracranial extension of the sinus tract. However, neither MRI nor CT images of the brain and cervical spine could explain the subtle UMN proprioceptive deficits in the cat, which would not be expected with cerebellar compression. One might speculate whether further malformative changes are present on a microscopic neuroparenchymal level.

Surgical management involved a combined bilateral mid-caudotentorial and limited right-sided rostrotentorial craniectomy, allowing complete excision of the sinus tract and neuroparenchymal decompression. Given the extent of intracranial involvement and the proximity to the cerebellum, the procedure was technically demanding. In particular, cerebellar signs such as ataxia, dysmetria, intention tremor or menace response deficits could have been postoperative concerns. Nevertheless, recovery was uncomplicated, with none of these signs occurring, and long-term follow-up at 28 months confirmed an excellent outcome with no recurrence. Complete removal of all components of the sinus tract, as achieved in this case, is crucial to prevent recurrence.

The rarity of intracranial dermoid sinuses in cats makes this case particularly noteworthy. Only a small number of dermoid sinuses with intracranial involvement have been described in small animal medicine, predominantly involving nasal sinuses (subtype c).^2,3,8,22–24^ Only five cases have previously been classified as subtype b (dermoid sinuses on the head, excluding the nose), all in dogs.^6,16–18,25^ Of these, only one was reported to have intracranial extension, expanding from the frontal region (type IVb), and was successfully treated surgically.^17^ In cats, a single case of a nasal dermoid cyst with intracranial extension was reported.^7^ In children, dermal sinuses with intracranial extension are also rare, and mostly described in the occipital region. Interestingly, they are usually associated with a deeper dermoid or epidermoid cyst, and infection occurs in about half the cases.^26^ To the authors’ knowledge, no non-nasal dermoid sinus with intracranial extension has previously been reported in a cat.

This case highlights the importance of including dermoid sinuses with potential intracranial extension as a differential diagnosis in young cats presenting with a mass on the head.

As dermoid sinuses can enlarge progressively and cause increasing neural compression over time, early recognition and prompt surgical intervention appear to offer the best prognosis. As stated for children, adequate cross-sectional diagnostic imaging and complete sinus removal are prerequisite.^26^

## Conclusions

This case contributes to the understanding of dermoid sinuses and their potential complications in small animal medicine. The successful outcome highlights the importance of thorough diagnostic imaging, confirmatory histopathology and meticulous surgical technique in achieving favourable long-term results.

## References

[1] Miller L Tobias K . Dermoid sinuses: Description, diagnosis, and treatment. Compend Contin Educ Vet 2003; 25: 295–300.

[2] Appelgrein C Hosgood G Reese SL. Computed tomography findings and surgical outcomes of dermoid sinuses: A case series. Aust Vet J 2016; 94: 461–466. 10.1111/avj.12525. 27800602

[3] Bailey TR Holmberg DL Yager JA. Nasal dermoid sinus in an American Cocker Spaniel. Can Vet J 2001; 42: 213–215. PMC1476468 11265192

[4] Barrios N Gómez M Mieres M , et al. Spinal dermoid sinus in a Dachshund with vertebral and thoracic limb malformations. BMC Vet Res 2014; 10. 10.1186/1746-6148-10-54. PMC3975874 24593884

[5] Booth MJ. Atypical dermoid sinus in a Chow Chow dog. J S Afr Vet Assoc 1998; 69: 102–104. 10.4102/jsava.v69i2.836. 9850515

[6] Bornard M. Bilateral parieto-occipital dermoid sinuses in a Rottweiler. J Small Anim Pract 2007; 48: 107–110. 10.1111/j.1748-5827.2006.00171.x. 17286665

[7] Bowens AL Ducoté JM Early PJ. What is your neurologic diagnosis? Type IV dermoid sinus (pilonidal sinus) in the region of the fourth thoracic vertebra. J Am Vet Med Assoc 2005; 227: 713–715. 10.2460/javma.2005.227.713. 16178391

[8] Brady S Bell E Courtman N , et al. Nasal dermoid cyst with intracranial extension in a cat. JFMS Open Rep 2019; 5. 10.1177/2055116919827404. PMC6376515 30792875

[9] Howard-Martin M Bowles MH. Intracranial dermoid cyst in a dog. J Am Vet Med Assoc 1988; 192: 215–216. 3350747

[10] Mann GE Stratton J. Dermoid sinus in the Rhodesian Ridgeback. J Small Anim Pract 1966; 7: 631–642. 10.1111/j.1748-5827.1966.tb04388.x. 5951011

[11] Rahal S Mortari AC Yamashita S , et al. Magnetic resonance imaging in the diagnosis of type 1 dermoid sinus in two Rhodesian Ridgeback dogs. Can Vet J 2008; 49: 871–876. PMC2519906 19043482

[12] Cornegliani L Ghibaudo G. A dermoid sinus in a Siberian Husky. Vet Dermatol 1999; 10: 47–49. 10.1046/j.1365-3164.1999.00110.x. 34644977

[13] Selcer EA Helman RG Selcer RR. Dermoid sinus in a Shih Tzu and a Boxer. J Am Anim Hosp Assoc 1984; 20: 634–636.

[14] Motta L Skerritt G Denk D , et al. Dermoid sinus type IV associated with spina bifida in a young Victorian Bulldog. Vet Rec 2012; 170. 10.1136/vr.100316. 22090157

[15] Fleming JM Platt SR Kent M , et al. Cervical dermoid sinus in a cat: Case presentation and review of the literature. J Feline Med Surg 2011; 13: 992–996. 10.1016/j.jfms.2011.08.003. PMC10832983 22079342

[16] Buijtels D Van Goethem B De Cock H , et al. Multiple dermoid sinus cysts on the head of a Miniature Schnauzer. Vlaams Diergeneeskd Tijdschr 2015; 84: 326–332. 10.21825/vdt.v84i6.16439.

[17] Danciu C Briola C Volckaert V , et al. Diagnosis and surgical management of a type IVb dermoid sinus in a Cavalier King Charles Spaniel. Vet Rec Case Rep 2023; 11. 10.1002/vrc2.786.

[18] Perazzi A Berlanda M Bucci M , et al. Multiple dermoid sinuses of type Vb and IIIb on the head of a Saint Bernard dog. Acta Vet Scand 2013; 55. 10.1186/1751-0147-55-62. PMC3846701 24006855

[19] Platt SR Radaelli ST McDonnell JJ. The prognostic value of the modified Glasgow Coma Scale in head trauma in dogs. J Vet Intern Med 2001; 15: 581–584. https://doi.org/10.1892/0891-6640(2001)015<0581:tpvotm>2.3.co;2. 10.1892/0891-6640(2001)015<0581:tpvotm>2.3.co;2 11817064

[20] Kiviranta AM Lappalainen AK Hagner K , et al. Dermoid sinus and spina bifida in three dogs and a cat. J Small Anim Pract 2011; 52: 319–324. 10.1111/j.1748-5827.2011.01062.x. 21627660

[21] Barrantes Murillo DF Rissi DR Wiener DJ , et al. Feline dermoid cyst: Retrospective case series and literature review. Vet Pathol 2025; 62: 293–299. 10.1177/030098582513745. 39968767

[22] Anderson DM White RAS . Nasal dermoid sinus cysts in the dog. Vet Surg 2002; 31: 303–308. 10.1053/jvet.2002.33597. 12094342

[23] Burrow RD. A nasal dermoid sinus in an English Bull Terrier. J Small Anim Pract 2004; 45: 572–574. 10.1111/j.1748-5827.2004.tb00207.x. 15553197

[24] Sturgeon C. Nasal dermoid sinus cyst in a Shih Tzu. Vet Rec 2008; 163: 219–220. 10.1136/vr.163.7.219. 18708657

[25] Schuldenfrei MD Affolter VK Outerbridge CA , et al. Locally extensive follicular hamartomas with concurrent follicular cysts and dermoid cysts on the head of a dog. Vet Dermatol 2022; 33: 459–462. 10.1111/vde.13084. 35661446

[26] Sáenz A Cicutti S Sánchez M , et al. Cranial dermal sinuses in pediatrics: Presentation, diagnosis, complications, and management. Experience at a tertiary care children’s hospital. Arch Argent Pediatr 2022; 120: 248–256 [article in English, Spanish]. 10.5546/aap.2022.eng.248. 35900951

